# The physical, psychological and social impact of long bone fractures on adults: A review

**DOI:** 10.4102/phcfm.v11i1.1908

**Published:** 2019-05-30

**Authors:** Sevani Singaram, Mergan Naidoo

**Affiliations:** 1 Discipline of Public Health Medicine, University of KwaZulu-Natal, Durban, South Africa; 2 Discipline of Family Medicine, University of KwaZulu-Natal, Durban, South Africa

**Keywords:** long bone fractures, impact, physical, psychological, occupational, financial, social

## Abstract

**Background:**

Long bone fractures are common injuries caused by trauma and are a common cause for referral to hospitals. Little consideration has been given to the impact of long bone fractures in adults despite the World Health Organization’s statement that such injuries cause substantial morbidity in low- and middle-income countries.

**Aim:**

This review targeted published studies conducted from 1990 to 2017 that examined the impact of long bone fractures on the psychological, social, financial, occupational and physical health of adults.

**Method:**

This scoping review involved a systematic literature search using key terms in Science Direct, Cochrane Library, BMJ Online, PubMed, Jstor, SpringerLink, Emerald Insight and Ebscohost Research databases and Google Scholar.

**Results:**

From a total of 297 publications, 19 met the inclusion criteria: four publications focused on the impact of fractures of the humerus, one publication looked at ulna fractures, six publications focused on distal radius fractures, five looked at femur fractures and three focused on tibial fractures.

**Conclusion:**

Long bone fractures have a considerable impact on many facets of a patient’s life. In some cases, the fracture prevents patients from working and meeting financial obligations. The injury limited previously normal social interactions and pre-injury functioning. Future research should examine the impact of long bone fractures in Africa, as there were very limited studies, which were identified.

## Introduction

Numerous studies and reports by the World Health Organisation indicate that injury is a substantial cause of morbidity and mortality in low- and middle-income countries (LMICs).^1^ The 2013 Global Burden of Disease and Injury study shows that road injuries are the seventh most common cause of disability adjusted life years.^2^ The Centres for Disease Control and Prevention indicates that fractures feature in the top-20 first-line diagnoses presenting to emergency departments.^3^ A fracture is ‘any loss in the continuity of bone’.^4^ Over the last several years, long bone fractures are becoming increasingly common, particularly because of road traffic injuries. More than 90% of injuries, particularly fractures of the extremity, occur in LMICs.^2^ Some studies have indicated that road traffic accidents cause 68.14% of fractures in some LMIC countries. Falls are also a serious public health problems worldwide because they can also cause re-injury. Some studies have demonstrated that falls have a prevalence of 21.8% and 35.1%.^5^ The burden of long bone fractures impacts society through the loss of productivity, the direct and indirect costs of treatment and the additional contribution to morbidity and mortality. The management and treatment of long bone fractures add significantly to the expenses of any health care system because of the cost of surgery, possible rehospitalisation and the physical rehabilitation of patients.^6^

Depressive symptoms such as catastrophic thinking, changes in appetite and sleep pattern are common after a fracture.^7^ As the standard of health and lifestyle improves in LMICs, one can expect that the older population, who are more prone to falls and fractures, will be greatly affected. Therefore, the burden is expected to rise substantially. We sought to answer the following question: what effect do long bone fractures have on the psychological, social, financial, occupational and physical health of patients?

## Methods

### Search strategy

Literature searches were conducted in Science Direct, Cochrane Library, BMJ Online, PubMed, Jstor, SpringerLink, Emerald Insight Ebscohost Research databases and Google Scholar to avoid missing other relevant articles not published in a journal. The reference lists of all chosen publications were also searched to source additional publications that may not have appeared in the search results. The search terms used were the following: impact of long bone fractures and/or psychological impact of long bone fractures and/or social impact of long bone fractures and/or financial impact of long bone fractures and/or occupational impact of long bone fractures and/or physical impact of long bone fractures and/or impact of humerus fractures and/or impact of radius fractures and/or impact of ulna fractures and/or impact of femur fractures, impact of tibia fractures and/or impact of fibula fractures.

Publications that were published in English only were included in the review. The inclusion criteria concentrated on studies that included participants aged 18 years and older who sustained one long bone fracture because of injury or pathology. For this review, only six long bones were included: the humerus, radius, ulna, femur, tibia or fibula. Both qualitative and quantitative studies published in English were included.

The study needed at least one outcome, that is, the psychological, social, financial, occupational or physical impact of the long bone fracture. Studies conducted before 1990, or those that did not reveal the age of participants or the name of the fractured bone, were excluded from the study.

To reduce bias, both authors (S.S. and M.N.) screened all titles and abstracts while being guided by the inclusion and exclusion criteria. Disagreement was resolved through discussion and final consensus. Publication bias was reduced by considering studies with limited sample sizes and studies with non-statistically significant results. Google Scholar was also searched to source grey literature.

### Review results

The study selection involved three steps:

In the first step, keywords and screening of titles were searched that returned 286 publications. Eleven publications were identified through a search of the reference lists. A total of 297 publications were included after exclusion criteria and duplicates were excluded.

The second step was the screening of the titles and abstracts, which was performed by the second reviewer that resulted in 267 publications being excluded. Five additional publications were excluded by the second reviewer after screening of the titles and abstracts.

Full-text screening was performed on 30 records. After full-text screening, five publications were excluded. This resulted in 25 publications having been assessed using the mixed-methods appraisal tool (MMAT) version 2018, a reliable critical appraisal tool that allows researchers to assess the methodological quality of publications because critical appraisal is an important aspect of scoping reviews.^8,9^ The MMAT is presented in Appendix 1. Only studies with a score of 50% or more were included. Six publications were excluded after being assessed using MMAT. Approximately 25 minutes was spent on the appraisal of each article. Finally, 19 eligible publications were included in the analysis.

### Data extraction and analysis

**FIGURE 1:**
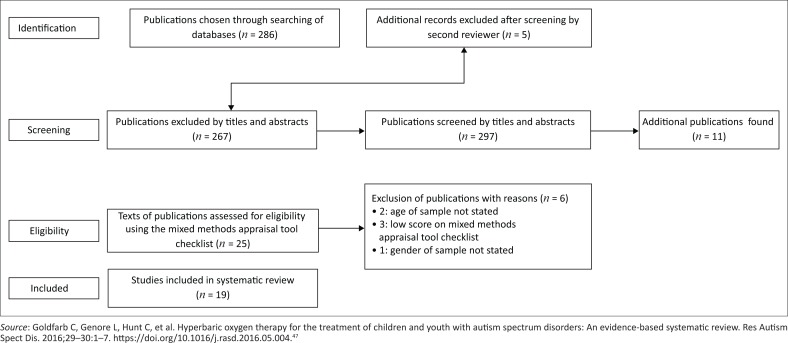
PRISMA 2009 flow diagram tool.

The PRISMA 2009 flow diagram tool was used to demonstrate the search process (see Figure 1). A standardised data extraction template was used to obtain data from the publications using the population, intervention, comparison, outcome and study design (PICOS) framework (see Tables 1 and 2). A meta-analysis was unsuitable because of the heterogeneity of the study variables and research designs. Content analysis was used to identify categories and report on findings. Content on the psychological, social, financial, occupational and physical impact was extracted from each publication.

**TABLE 1:** Upper extremity long bone fractures.

Research question	Population	Intervention	Comparator	Outcome	Study design	Reference
To investigate the physical outcome of proximal humerus fractures	Proximal humerus fractures Sample size = 104 80% female 20% male Mean age = 63 years	Standardised physical therapy regime at an average of 13 days after the injury	Nil	The duration of follow-up averaged 41 months. Functional recovery averaged 94%. Forty-six percent had 100% functioning recovery. At final follow-up, the percentage of positive recovery was greater (*p* < 0.01).	Qualitative descriptive, review of charts and physical examination	^10^
To compare immediate mobilisation with conventional immobilisation after a proximal humerus fracture	Proximal humerus fractures Sample size = 74 Mean age (early mobilisation group) = 63.2 years 65% female 25% male Conventional Treatment group 63.4 years 81% female 19% male	Early mobilisation of fracture	Patients that underwent conventional treatment	Immediate mobilisation offers better chance for a full recovery of shoulder functional status. At 3 months, the early mobilisation group reported less pain compared to those treated with the conventional treatment (between group difference, 15.7 %–95 % confidence interval, 0.52–30.8) (*p* = 0.04).	Randomised control trial	^11^
To investigate the physical outcome of a two-part proximal humerus fracture	Proximal humerus fractures Sample size = 50 80% female 20% male Mean age = 75 years	Locking plate	Nil	A comparison of functional outcome for patients aged under 70 and those over 70 years showed better values for the younger age group. Despite the overall acceptable functional outcome after treatment with a locking plate, many patients reported that the fracture had a negative effect on their quality of life. After the fracture, the disabilities of the arm, shoulder and hand (DASH) scores and constant scores were significantly lower (*p* < 0.01).	Prospective cohort	^12^
To investigate the physical outcome after a proximal humerus fracture	Proximal humerus fractures Sample size = 158 Male: 32 Female: 126 Mean age = 78 years	Open reduction with internal fixation or arthroplasty	Nil	There was substantial mortality in patients with a proximal humerus fracture. Surviving patients have persistent symptoms that can be predicted as early as 1 year. There was a correlation between poor health and fracture outcomes (*p* = 0.01).	Prospective cohort	^13^
To investigate the physical outcome after a distal radius fracture	Distal radius fractures Sample size = 250 34% male 66% female Range of age = 18 to >65 years	Physiotherapy	Nil	Fifty percent of patients found physiotherapy helpful, 27% found quite helpful, 15% found moderately helpful, 5% found slightly helpful and 2% found not helpful at all. Age and gender did not contribute to differences in functional scores.	Prospective cohort	^14^
To investigate the functional outcomes of distal radius fractures in elderly patients	Distal radius fractures Female: 21 Male: 1 Mean age = 69.4 years	Closed and/or per-cutaneous reduction and pinning, and internal fixation with plate or external fixation	Nil	Most patients achieved excellent functional results regardless of variable residual deformities. Some patients showed a decrease in grip strength and had arthritis. At the last check-up, functional outcomes were regarded as excellent.	Retrospective review	^15^
To investigate disability after a distal radius fracture	Distal radius fracture Sample size = 129 68% female 32% male Mean age 50 years	Reduction and fixation	Nil	Symptoms subsidised within the first 2 months and most recovery occurred within 6 months. A small fraction of patients reported that symptoms continued for 1 year after the fracture.	Prospective cohort	^16^
To investigate time lost from work after a distal radius fracture	Distal radius fractures Sample size = 227 42% male 58% female Mean age = 43.8 years	Standard care	Nil	The average number of weeks lost from work was 9.2. Twenty-one percent reported no time lost from work. Patients with greater disability are at risk of prolonged work loss.	Prospective cohort	^17^
To investigate the impact of distal radius fractures on quality of life	Distal radius fractures 160 patients and 169 age and sex matched controls Mean age of patients = 67 years Mean age of control = 66 years	Standard care	Patients with no distal radius fracture	After 1 year, no differences were found in Health-Related Quality Of Life (assessed as physical health and mental health) compared to before the fracture in the patient group. Those with distal radius fractures and controls reported a reduced general quality of life (GQOL) 1 year later (*p* < 0.001).	Prospective longitudinal	^18^
To investigate if malunion affects the functional outcome of distal radius fractures	Distal radius fractures Sample size = 52 Females: 51 Male: 1 Mean age = 83.1 years	Fracture manipulation or surgery	Distal radius fracture patients without malunion	Malunion of the distal radius does not influence the functional outcome of independent elderly patients. No differences were found in activities of daily living (*p* = 0.28), wrist pain (*p* = 0.14), grip strength (*p* = 0.31) or range of movement (*p* = 0.41).	Retrospective cohort	^19^
To compare the results of operative and non-operative treatment of ulna shaft fractures	Ulna shaft fractures Sample size = 70 45.5% male 54.5% female Mean age = 44.6 years	Reduction with internal fixation	Patients with non-operative treatment in isolated ulna shaft fractures	Non-operative treatment of displaced fractures produces a higher risk of complications. The fracture characteristics determine patient outcome. Age, gender and treatment did not relate or contribute to clinical or functional results. Fracture angulation greater than 8^o^ correlated with not returning to the previous level of activity (*p* = 0.001).	Retrospective case control	^20^

**TABLE 2:** Lower extremity long bone fractures.

Main aim	Population	Intervention	Comparator	Outcome	Setting	Reference
To investigate the physical outcome of atypical and typical femoral fractures	Femur fractures Sample size = 10 90% female 10% male Mean age = 78.1 years	Nine patients had their fractures fixed with an intramedullary nail. Eight had taken bisphosphonate	Patients with atypical femoral fractures	The levels of mobility at discharge (*p* = 0.26) and at 3 months (*p* = 0.47) were different between those with atypical and typical femoral fractures.	Retrospective matched cohort	^21^
To investigate the physical outcome of distal femur fractures in geriatrics	Distal femur fractures Sample size = 43 4.7% male 95.3% female Mean age = 80 years	Less invasive stabilisation system plate	Nil	Five years after the fracture, only 18% could walk unaided. In comparison to other geriatric fracture patients, patients with femur fractures face a higher risk of mortality.	Cohort with functional long-term follow-up examination	^22^
To investigate the comparison of femoral functional recovery after plate and nail fixation	Femoral intertrochanteric fracture Sample size = 18 Male: 16 Female: 2 Mean age = 79.7 years	Femur surgery using plate fixation	Femur surgery using nail fixation	The results suggested that nail fixation may provide a more rapid recovery of activities of daily living after surgery (*p* = 0.03), although, plate fixation provided greater range of flexion (*p* = 0.04).	Controlled clinical trial	^23^
To investigate the effect of rehabilitation on physical outcome after a femur fracture	Femoral neck or intertrochanteric fractures Sample size = 609 Female: 490 Male: 119 28.6% (> 85 years) 71.4% (< 85 years) Mean age of geriatric patients not revealed	Acute inpatient rehabilitation	Patients who did not receive rehabilitation	No significant difference in level of recovery at discharge was noted between patients who underwent rehabilitation and those who did not (*p* < 0.01).	Prospective cohort	^24^
To investigate quality of life after a femoral neck fracture	Femoral neck fractures Sample size = 90 Mean age = 80 years	Internal fixation	Nil	There was a substantial decrease in quality of life after the fracture according to the EQ-5D questionnaire. The results were considered significant (*p* < 0.05).	Prospective	^25^
To investigate the long-term complication of tibial shaft fractures	Tibial shaft fractures Sample size = 572 19% female 81% male Mean age = 35 years	Conservative treatment of fracture	Patients without a fracture	Patients with tibial shaft fractures are more likely to suffer pain and osteoarthritis (odds ratio 1.23; 95% confidence interval [CI] 1.00, 1.51).	Retrospective matched cohort	^26^
To describe the impact of an open tibial fracture	Open tibial fractures Sample size = 9 Males: 6 Females: 3 Mean age = 44 years	Circular external fixation or intramedullary nail	Nil	The mean injury to interview interval was 2.3 years. Pain, changes in sleep patterns and fear of re-injury were reported. Although health care professionals considered patients to have recovered from the fracture, patients did not return to pre-injury mortality.	Qualitative descriptive	^27^
To describe the physical and occupational impact of tibial fractures	Distal Tibia fractures Sample size = 25 76% male 24% female Mean age = 46.3 years	Standard care	Nil	Forty-eight percent of patients stated that their job involved climbing while 84% said that their job required prolonged standing. The mean return time to work was 24 months. Those with higher education and white-collar jobs returned to work sooner (*p* = 0.001).	Retrospective review	^28^

EQ-5D, EuroQol five-dimension scale – an instrument used for measuring quality of life.

### Ethical considerations

This review is part of a PhD thesis, which has been reviewed by the Biomedical Research Ethics Committee, under the protocol reference number BE 583/16. The title of the thesis is ‘The perceived psychological, social, financial, occupational and physical impact of long bone fractures in adults in KwaZulu-Natal’. The study enrolled 821 research participants from nine hospitals. To our knowledge, there is no other study investigating this topic. The purpose of this review is to gather information on this topic.

## Results

### Study characteristics

The average age of participants in all studies was 63.7 years. The earliest study was conducted in 1997 and the latest study was in 2016. Three studies were set in Japan, three in Canada, one in Australia, one in France, one in Austria, one in Norway, four in the United Kingdom, and three in the United States of America, two of which were in New York City and one in Grand Rapids. Orthopaedic research regarding the psychological, social, financial and occupational impact of long bone fractures in LMICs has unfortunately been very limited because these countries have mainly focused on infectious and nutritional illnesses.^29–31^

### Extent and trend of the studies

The sample size ranged from 9 to 609 respondents per study. In some studies, the respondents had a surgical orthopaedic intervention. Four studies focused on fractures of the humerus, one on ulna fractures, six on distal radius fractures, five on femur fractures and three on tibial fractures. Most of the studies focused on distal radius and femur fractures. Three studies focused on the physical impact of proximal humerus fractures, one on the physical and occupational impact of ulna fractures, two on the physical and occupational impact of long bone fractures, two on the physical impact of distal radius fractures and one on the physical and psychological impact of distal radius fractures. Four studies focused on the physical impact of femoral fractures; one on the physical and social impact of femur fractures; one on the financial, occupational and social impact of tibia fractures; one on the physical impact of tibia fractures; and one on the physical, financial, social, psychological and occupational impact of tibia fractures. There were very few studies on the impact of fibula fractures, but none of them met the criteria for this systematic review. Most of the studies focused on the physical impact of the long bone fractures.

#### The biopsychosocial approach to long bone fractures

**FIGURE 2:**
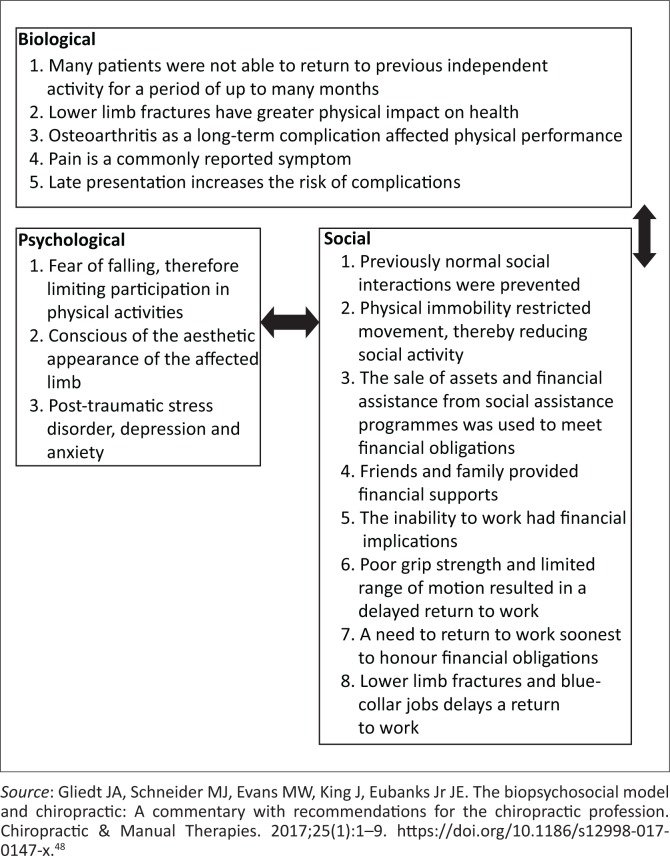
The biopsychosocial model.

The World Health Organization states that ‘health is a complete state of physical, mental and social well-being and not merely the absence of disease or infirmity’.^32,33^ The biopsychosocial model (BPSM) offers a broader and holistic approach for healthcare professionals to understand human behaviour, disease and infirmities.^32,33^ The biopsychosocial approach should be applied after a patient sustains a fracture and this may aid in addressing other factors that might influence an individual’s recovery. The BPSM comprises three dimensions that can be used to assess post-fracture outcomes: biological, psychological and social factors. The biological dimension deals with the physical impact of the fracture.^33^ The psychological dimension comprises the psychodynamic factors affecting patients after the fracture and the social dimension examines the external influences such as support from family and friends, financial influences and possible changes to job or loss of income after the fracture. Research from high-income countries suggests that timely and adequate treatment leads to quicker recovery. Various authors have suggested that health care resources and finances of the patient also influence a patient’s recovery.^34^ To accurately understand the impact of long bone fractures, the BPSM was adopted as the analytical framework. The literature supports this framework as fractures affect the biological, psychological and social aspects of a patient’s life (see Figure 2).

The results will therefore be presented under three headings, namely, psychological and social, financial and occupational and physical impact of long bone fractures.

#### Psychological and social impact of long bone fractures

Psychological sequelae are common after long bone fractures and include post-traumatic stress disorder, depression and anxiety.^35^ Depression is common following injury and may adversely affect clinical outcomes. Further to this, a failure to return to the level of functioning before the fracture may cause symptoms of depression.^35^ Trickett and colleagues’^27^ qualitative study described how patients felt after an open tibial fracture. All patients were fearful of falling and were conscious of the aesthetic appearance of the fractured limb. Two patients described using alcohol as a means of coping with the circumstances surrounding the injury. In contrast, MacDermid and colleagues^14^ found that distal radius fractures had minimal effect on the patient’s psychological state. In a 2017 study by Grenier et al.^36^ it was found that the presence of a fear of falling causes dysfunction in the neural networks connected to motor imagery abilities, and therefore these patients are more susceptible to re-injury. Fearful patients may not comply with the physical rehabilitation which may reduce one’s ability to complete activities of daily living leading to an increase in patient dependency. Glover and colleagues^37^ advise that the most effective approach to managing fear of falls is psychological and physical intervention. Kammerlander et al.^22^ found that 23% of patients were physically unable to leave their home after sustaining a distal femoral fracture and as a result were not able to socialise. In 2012, Trickett and colleagues^27^ also confirmed that a tibial fracture prevented previously normal social interactions. This lack of social support may contribute to injury reoccurrence, rehospitalisation and higher personal and societal health care costs. The use of caregivers with a health care background is a useful way of lending emotional support to patients to reach their recovery goals.^38^

#### Financial and occupational impact of long bone fractures

Volgas and colleagues^28^ examined the financial implications of tibial fractures after 6–20 months and found that 29% of patients returned to work at the anticipated time of recovery, 36.8% reported selling possessions to pay their expenses and 42.1% used social welfare initiatives. It was noted that all patients in white-collar jobs returned to work sooner than others, whereas only 14.3% of blue-collar workers returned to work at the last follow-up visit. Forty-two percent of patients used friends and family as a source of financial aid. Only 29.2% of patients returned to work at a mean follow-up of 11.8 months.^28^ Trickett and colleagues^27^ described how the inability to work, following a tibial fracture, led to financial implications. Larsen and colleagues^39^ suggest that fractures of the lower extremity limit positive results, such as returning to work and performing activities of daily living independently. Returning to work is regarded as an important outcome in orthopaedic treatment. Mackenzie et al. state that patients who return to work sooner are usually younger and have higher social support.^40^ Coulibaly and colleagues^20^ found that in isolated ulna shaft fractures, 87% of patients returned to a pre-fracture level of activity or work. MacDermid and colleagues^17^ described the days lost from work following a distal radius fracture. Patients with a limited range of motion were less likely to return to work. The average number of weeks lost from work was 9.5. Trickett and colleagues^27^ indicated that many non-retired patients wanted to return to work soon after a tibial fracture because of the inability to financially provide for themselves and to return to normality. One patient described how he had to adapt his duties and hours of work after the injury to remain involved at work.

Sluys and colleagues^41^ confirm that patients with fractures of the lower extremity return to work later compared to those with fractures of the upper extremity.

#### Physical impact of long bone fractures

Pain is one of the main symptoms of a fracture that causes the patient to seek medical attention after the initial injury. Swelling, reduced mobility of the affected joint and deformity of the limb are common causes of pain. Some fractures are associated with less pain, resulting in some patients presenting late for treatment.^4,42^ The possible implications of not receiving appropriate and timely treatment after sustaining a fracture are malunion, avascular necrosis, fat embolism syndrome and prolonged immobility.^4^ Fractures with injury extension to the soft tissue, nerve or vasculature may cause complex regional pain syndrome.^43^ An important consideration in the initial management of fractures is a clinical pathway for pain management. Pain is often dependent on a host of factors that may or may not be because of the severity of the fracture. Good pain management facilitates patient comfort and reduces anxiety, allowing for a better orthopaedic assessment and compliance with the rehabilitation plan.^4,42^

Upper extremity long bone fractures often affect activities such as personal hygiene, eating and writing, especially if the dominant limb is fractured. Frail geriatrics may require additional support during the period of immobilisation. Swelling following fracture is a common contributor of pain that is exacerbated by a tight plaster cast, but this could be managed by elevation of the arm and monitoring the swelling. Lower limb long bone fractures often result in an inability to carry out activities of daily living and usually affect employment, especially if the patient requires mobility in their occupation. Many patients with lower limb fractures require hospitalisation.^4,39,42^

Osteoarthritis is a long-term complication of fractures, especially in the lower limbs, and can lead to chronic pain. Approximately 12% of all patients seeking treatment for symptomatic arthritis reported previous injury to the joint.^44^ There can be significant joint degeneration after severe joint injury such as an articular fracture.^4^

## Discussion

This scoping review aimed to investigate the impact of long bone fractures on the psychological, social, financial, occupational and physical health of adults. The psychological impact of long bone fractures includes fear of falling and, therefore, limiting participation in physical activities, being conscious of the aesthetic appearance of the affected limb, post-traumatic stress disorder, acute stress disorder, depression and anxiety. Fractures can also impact the patient’s social life because of limited mobility that compromises social activity. Patients reported selling possessions and making use of social assistance programmes to pay for expenses incurred as a direct result of the fracture. Some used friends and family as a source of financial aid. Physical discomfort, pain, immobilisation, deformity and the nature of some fractures result in prolonged absence from work. Patients with limited range of motion were less likely to return to work without vigorous physical rehabilitation. Those who were gainfully employed expressed a need to return to work to honour their financial obligations and as a signal of the gradual return to normality.

The fracture prevented patients from returning to previous level of activity for many months, with pain being the common reason for this.

The public health impact of fractures includes increases in physical impairments and psychological symptoms of fear of re-injury and post-traumatic stress disorder.^45^ Further to this, equal access to orthopaedic care and surgery in many African countries remains a challenge because of the shortage of health care practitioners and limited resources.

As many countries in Africa are already struggling with poverty, human immunodeficiency virus and a shortage of health care workers, sustaining an injury can place an added burden on patients.^46^ Another important public health consideration is that in some instances, a proximal femur fracture places the individual at a high risk of sustaining another fracture. This may create additional costs to the individual because of possible rehospitalisation and loss of work.^45^

### Study limitations

This review only included studies published in English as it would have been costly and time-consuming to enlist the services of many translators for various languages. This review shows the need for studies that include younger participants. We acknowledge that we may have missed important evidence because of our inclusion and exclusion criteria.

### Implications and recommendations

According to the World Health Organization, 90% of injuries occur in LMICs, such as those in Africa. Therefore, there is a need for more studies to assess the financial, occupational, social and psychological impacts of long bone fractures because there are many studies that only have documented the physical impact of long bone fractures, particularly in Africa. The impact of fractures in LMICs could be magnified because of poorly developed trauma care and limited social infrastructure.

## Conclusion

Long bone fractures have a considerable impact on the physical outcome of patients. In some cases, the fracture prevents patients from working and meeting financial obligations. In many cases, the injury limited previously normal social interactions and pre-injury functioning. The findings should be considered while training health workers and providing counselling to orthopaedic patients.

## Appendix

**APPENDIX 1:** Mixed methods appraisal tool, version 2018.

Category of study designs	Methodological quality criteria	Responses			
		Yes	No	Can’t tell	Comments
Screening questions (for all types)	S1. Are there clear research questions?				
	S2. Do the collected data allow to address the research questions?				
	*Further appraisal may not be feasible or appropriate when the answer is ‘No’ or ‘Can’t tell’ to one or both screening questions.*				
1. Qualitative	1.1. Is the qualitative approach appropriate to answer the research question?				
	1.2. Are the qualitative data collection methods adequate to address the research question?				
	1.3. Are the findings adequately derived from the data?				
	1.4. Is the interpretation of results sufficiently substantiated by data?				
	1.5. Is there coherence between qualitative data sources, collection, analysis and interpretation?				
2. Quantitative randomised controlled trials	2.1. Is randomisation appropriately performed?				
	2.2. Are the groups comparable at baseline?				
	2.3. Are there complete outcome data?				
	2.4. Are outcome assessors blinded to the intervention provided?				
	2.5 Did the participants adhere to the assigned intervention?				
3. Quantitative non-randomised	3.1. Are the participants representative of the target population?				
	3.2. Are measurements appropriate regarding both the outcome and intervention (or exposure)?				
	3.3. Are there complete outcome data?				
	3.4. Are the confounders accounted for in the design and analysis?				
	3.5. During the study period, is the intervention administered (or exposure occurred) as intended?				
4. Quantitative descriptive	4.1. Is the sampling strategy relevant to address the research question?				
	4.2. Is the sample representative of the target population?				
	4.3. Are the measurements appropriate?				
	4.4. Is the risk of non-response bias low?				
	4.5. Is the statistical analysis appropriate to answer the research question?				
5. Mixed methods	5.1. Is there an adequate rationale for using a mixed-methods design to address the research question?				
	5.2. Are the different components of the study effectively integrated to answer the research question?				
	5.3. Are the outputs of the integration of qualitative and quantitative components adequately interpreted?				
	5.4. Are divergences and inconsistencies between quantitative and qualitative results adequately addressed?				
	5.5. Do the different components of the study adhere to the quality criteria of each tradition of the methods involved?				

*Source*: Hong QN, Bartlett G, Vedel I, et al. The Mixed Methods Appraisal Tool (MMAT) version 2018 for information professionals and researchers. Education for Information. 2018;34(4):285–291. https://doi.org/10.3233/EFI-180221.^49^
